# Circulating memory T follicular helper subsets, Tfh2 and Tfh17, participate in the pathogenesis of Guillain-Barré syndrome

**DOI:** 10.1038/srep20963

**Published:** 2016-02-11

**Authors:** Yuanyuan Che, Jinpeng Qiu, Tao Jin, Fei Yin, Man Li, Yanfang Jiang

**Affiliations:** 1 Genetic Diagnosis Center, The First Hospital of Jilin University, Changchun 130021, China; 2 Key Laboratory of Zoonoses Research, Ministry of Education, The First Hospital of Jilin University, Changchun 130021, China; 3 Jiangsu Co-innovation Center for Prevention and Control of Important Animal Infectious Diseases and Zoonoses, Yangzhou 225009, China; 4 Department of Neurology, The Second Hospital of Jilin University, Changchun 130021, China

## Abstract

Circulating memory T follicular helper subsets, Tfh2 and Tfh17 are found to be aberrantly regulated in many autoimmune diseases. However, their roles in the pathogenesis of GBS are still unclear. This study examined the phenotype, distribution, clinical relevance and potential function of Tfh2 and Tfh17 in 36 GBS patients (including 24 AMAN and 12 AIDP patients). We found that the absolute counts of total memory Tfh cells were significantly increased in AMAN, while no significant difference in AIDP compared with HC. Furthermore, the levels of the three subsets of memory Tfh cells, Tfh1, Tfh2 and Tfh17, were differentially altered in AMAN. The absolute counts of Tfh1, Tfh2 and Tfh17 were all increased to a higher level in AMAN. The ratio of (Tfh2+Tfh17)/Tfh1 and the percentages of ICOS^+^ cells in Tfh2 and Tfh17 cells were greater in AMAN when compared to AIDP and HC, and the former had a positive correlation with the severity of both AMAN and AIDP. Conversely, the percentages of PD1^+^ cells in Tfh2 and Tfh17 cells were lower in AMAN than in HC. Therefore, circulating memory Tfh2 and Tfh17 cells might promote the autoantibody-related immune response and serve as useful markers to evaluate the progression of AMAN.

Guillain-Barré syndrome (GBS), including its various subgroups, is an acute autoimmune mediated inflammatory demyelinating disease that affects the peripheral nervous system (PNS). Though pathological changes in GBS are known to include segmental demyelination of peripheral nerves associated with infiltration of T lymphocytes and macrophages^1^, considerable knowledge gaps persist regarding the precise immunopathogenesis of nerve damage. It has been hypothesized that autoreactive CD4^+^T helper-cell-mediated immune damage in parallel with increased cytokine expression contributes to the inflammatory process of GBS, and in the animal model, for experimental autoimmune neuritis (EAN)^2^,^3^,^4^,^5^,^6^,^7^,^8^. Recent studies have implicated aberrant humoral immunity as also being involved in the pathogenesis of GBS, including anti-ganglioside antibody production in cerebrospinal fluid (CSF)^9^; and its pivotal role in motor nerve terminal injury in a mouse model^10^.

Evidence in favor of involvement of humoral immunity in the pathogenesis of GBS include studies that have reported deposition of immunoglobulin G and complement activation products on the axolemma of motor fibers^11^; and the presence of myelin specific plasmablasts and B cell expansion in spontaneous autoimmune polyneuropathy (SAP), an animal model of GBS, and depletion of these cells in response to anti-CD19 monoclonal antibody (mAb) treatment, leading to attenuation of disease severity^12^.

The current report highlights the differences in the clinical characteristic and pathogenesis of two distinct forms of GBS, namely acute inflammatory demyelinating polyradiculoneuropathy (AIDP) and acute motor axonal neuropathy (AMAN). The former is characterized by mutiple segmental demyelinative foci throughout the PNS, the latter displays primary axonal degeneration particularly accentuated at the spinal nerve roots. The key etiologic factor of AIDP may be the T cell-mediated autoimmunity against myelin antigens on the Schwann cell membrane, and that in AMAN may be an autoantibody-mediated attack on the axolemma at the Ranvier nodes^13^.

Most of the mild cases of GBS respond to conventional treatment with high-dose intravenous immunoglobulin (IVIg). However, a subset of patients with severe disease tends to respond poorly to IVIg therapy, and experience a rapid progression of respiratory muscle paralysis and even death. Moreover, ganglioside-associated GBS, which mainly involves axonal injury, is known to be associated with severe manifestations and poorer short-term prognosis^14^. Therefore, a thorough comprehension of the immunological pathogenesis, especially T-B cell interaction in GBS, will be helpful for developing more effective GBS immunotherapies.

Human peripheral blood CD4^+^CXCR5^+^CD45RA^−^T cells, are known as “circulating memory” follicular helper CD4^+^T (Tfh) cells^15^,^16^. As reported, human circulating memory Tfh cells share phenotypic and functional properties with Tfh cells residing in germinal center (GC Tfh cells), such as enhanced expression of CXC-chemokine receptor 5 (CXCR5), stimulation of B-cell maturation, terminal differentiation of B cells into antibody-producing plasma cells, and isotype switching^15^,^16^. The specialized molecular markers of memory Tfh cells include inducible T-cell co-stimulator (ICOS), programmed death-1 (PD-1), CD40 ligand (CD40L), transcription factor B cell lymphoma 6 (Bcl-6), SAP (signaling lymphocytic activation molecule associated protein) and interleukin-21 (IL-21)^17^.

The circulating human memory Tfh cells have hitherto been divided into three subsets: Tfh1 (CXCR3^+^CCR6^−^), Tfh2 (CXCR3^−^CCR6^−^) and Tfh17 (CXCR3^−^CCR6^+^)^16^. Previous reports have suggested that a skewed distribution of circulating memory Tfh cell subsets contributes to the pathogenesis of some autoimmune diseases such as primary sjögren’s syndrome, wherein there are higher levels of Tfh17^18^; and juvenile dermatomyositis, where there are higher levels of Tfh2 and Tfh17^16^. Co-culture experiments have demonstrated that Tfh2 and Tfh17, but not Tfh1, could efficiently induce naive B cells to produce immunoglobulins via secretion of interleukin-21 (IL-21)^16^. In a recent study, circulating memory Tfh cells were reported as playing a crucial role in the immunopathogenesis of certain neurologic autoimmune diseases such as multiple sclerosis^19^ and myasthenia gravis^20^, in which the role of humoral immunity is generally recognized. However, little is known about the distribution and function of circulating memory Tfh cell subsets in GBS patients.

In the present study, the frequency and the phenotypic characterization of circulating memory Tfh cells was assessed to examine their role in the immunopathogenesis of GBS.

## Materials and Methods:

### Patients

Thirty six newly diagnosed cases of GBS who fulfilled the international diagnostic criteria for this disease^21^ were enrolled in the study. Disease severity was evaluated using the Hughes Functional Grading Scale (HFGS) score, and the Medical Research Council (MRC) sum score. The former is a widely recognized scoring system for assessing the functional status of GBS patients^22^, and the latter is used for evaluation of the neurologic function of six bilateral muscles in the arms and legs^23^. Eighteen age- and sex-matched healthy individuals served as controls in this study. Patients with any other underlying immune-mediated disorder or chronic inflammatory demyelinating polyradiculoneuropathy were excluded from the study. Routine CSF examination and electromyography were performed within 2 weeks of onset of symptoms. Venous blood was sampled during the acute phase (1–14 days after onset) of GBS. After treatment with IVIg, single dose of 0.4g/kg body weight per day for 5 consecutive days, blood samples of the 7 patients with more serious clinical signs (HFGSs ≥4) were also collected during the plateau phases (15–32 days after onset). CSF and serum samples from cases were collected and stored at −80 °C for further analysis. Written informed consent was obtained from all study subjects. Ethical approval of the study protocol was obtained from the Ethics Committee of the First Hospital of Jilin University. All procedures were carried out in accordance with the approved guidelines. All experimental protocols were approved by the above authority.

### Cell isolation and flow cytometry

Peripheral blood mononuclear cells (PBMCs) were isolated by density-gradient centrifugation using Ficoll-Paque Plus (Amersham Biosciences, Buckinghamshire, UK). The cells were then resuspended to 1 × 10^6^ per mL in RPMI-1640 culture medium (Invitrogen, Carlsbad, CA, USA) containing 10% fetal calf serum, and stained with the following monoclonal antibodies: antihuman CD3-BV510, antihuman CD4-APC-H7, antihuman CXCR5-PerCP-Cy5.5, antihuman CXCR3-PE-Cy7, antihuman CCR6-PE, antihuman CD45RA-PE-CF594, antihuman ICOS-BV421, antihuman PD1-FITC, antihuman CD27-PE, antihuman CD20-APC-H7, antihuman CD38-PerCP-Cy5.5, antihuman CD19-PE-cy7 or isotype-matched control IgG (BD PharMingen San Diego, CA, U.S.). After washing twice with phosphate-buffered saline (containing 0.1% (w/v) NaN3), the samples were analyzed using FACS Aria II (BD Sciences, San Jose, USA); at least 50,000 events per sample were analyzed using FlowJo software (v7.6.2). The absolute count of cells was calculated on the basis of the cell proportion multiplied by the absolute number of lymphocytes.

### Cell sorting and Tfh/B co-culture

For sorting circulating memory Tfh subsets and B cells by flow cytometry, freshly isolated PBMC from GBS patients and healthy controls were stained with antihuman CD4-APC-H7, antihuman CXCR5-PerCP-Cy5.5, antihuman CXCR3-PE-Cy7, antihuman CCR6-PE, antihuman CD45RA-PE-CF594, and antihuman CD19-FITC. Sorted cells were divided into two parts, one containing a mixture of Tfh2 and Tfh17 (CD4^+^CXCR5^+^CD45RA^−^CXCR3^−^) cells, and the other containing the B (CD19^+^) cells. Tfh2 and Tfh17 cells (3 × 10^5^ cells per well) were co-cultured for 6 days with allogenic B cells (1 × 10^5^ cells/well) in U-bottom 48-well plates containing complete RPMI 1640 medium (Lonza, Alendale, NJ, USA) supplemented with 10% heat-inactivated FBS. Parallel plates were tested in triplicate. Concentrations of IgG, IgA and IgM were measured in co-culture supernatants by Cytometric Bead Array, and phenotypic characterization of B cells performed by flow cytometry.

### Cytometric Bead Arrays (CBA) of immunoglobulin levels

Co-culture supernatants were collected and stored at −80 °C until further testing. The concentrations of IgG, IgA and IgM were measured by CBA Human Immunoglobulin Master Buffer Kit (BD Biosciences San Jose, CA, US), as per the procedure recommended by the manufacturer.

### Enzyme-linked immunosorbent assay for IL-21 levels

The levels of IL-21 were measured in serum, CSF and co-culture supernatants by ELISA (Multi Sciences Bio-Technology Co, Hangzhou, China) as per the manufacturer’s instructions. The detection limit for human IL-21 was 11.99 pg/mL.

### Statistical analyses

Data are expressed as median (range) or as individual mean values. Inter-group differences were evaluated by F-test followed by the two-tailed Student’s, or paired *t*-test. A Wilcoxon signed-ranks test was performed for variables that did not exhibit Gaussian distribution. Pearson correlation coefficient was determined for evaluating the observed correlations. A two-side *P* value of <0.05 was considered statistically significant.

## Results

A total of 36 cases of GBS were enrolled in the study, out of which 24 and 12 patients were diagnosed with AMAN and AIDP, respectively. None of the patients had received immunosuppressive or immunomodulatory drugs in the preceding three months. Baseline characteristics of patients and controls are summarised in Table 1; clinical characteristics and laboratory data of GBS patients are summarized in Tables 2, 3 and 4.

### High absolute counts and skewed distribution of circulating memory Tfh cell subsets in AMAN patients

**Figure 1:**
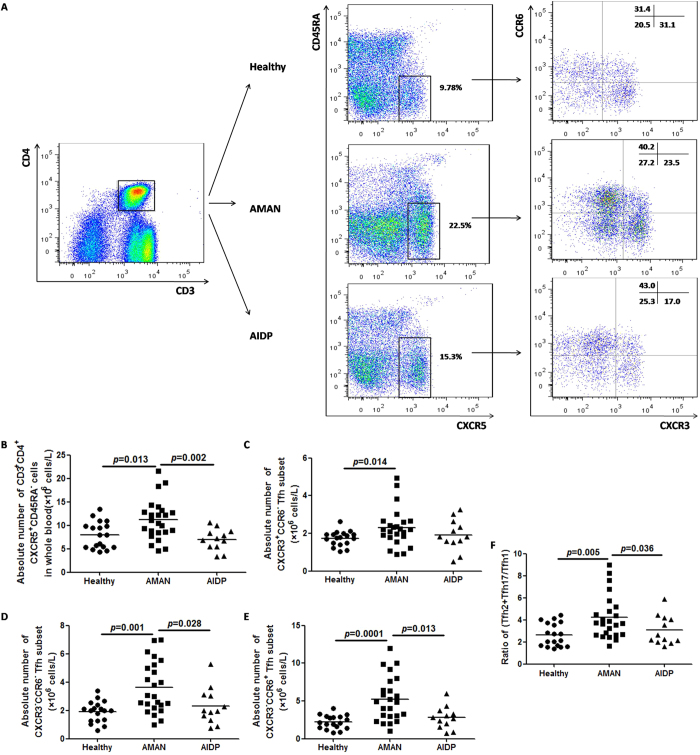
Altered expression of circulating memory Tfh cell subsets (Tfh1, Tfh2 and Tfh17) in Guillain-Barré syndrome (GBS) patients (with high frequency and skewed distribution). (**A**) Fluorescence Activated Cell Sorting (FACS) analyses of circulating memory Tfh cells. Peripheral mononuclear cells (PBMCs) were stained with anti-CD3/4, anti-CD45RA/CXCR5 and anti-CXCR3/CCR6. The cells were gated initially on CD3+CD4+ T helper cells (top left). Subsequently, the frequency of circulating memory Tfh cells (CD45RA-CXCR5+) on Th (CD3+CD4+), and the Tfh1 (CXCR3+CCR6-), Tfh2 (CXCR3-CCR6-), or Tfh17 (CXCR3-CCR6+) subsets, respectively, on total Tfh were analyzed by flow cytometry. At least 30,000 events were analyzed for each sample. Data are representative of different groups of samples from at least two independent experiments. (**B**–**F**) Circulating memory Tfh cell subsets from GBS patients (N = 36), including subgroups of acute inflammatory demyelinating polyradiculoneuropathy patients (AIDP, N = 12) and acute motor axonal neuropathy patients (AMAN, N = 24); and healthy controls (HC, N = 18) were analyzed by flow cytometry *ex vivo*. Data are expressed as absolute number of total Tfh cells and the three subsets in whole blood, and expressed as cells/L of individual samples from at least two separate experiments; then compared with the ratio of (Tfh2+Tfh17)/Tfh1. Data analysis using the Mann Whitney test. The horizontal lines represent the median values.

As shown in the representative flow cytometry dot plots (Fig. 1a), the frequency of circulating memory Tfh cells in AMAN group was significantly higher than that in the HC and AIDP groups (Fig. 1b). As to the three subsets, the counts of Tfh1, Tfh2 and Tfh17 cells were all raised to a higher level in the AMAN group, but only the percentages of Tfh2 and Tfh17 cells were higher in AMAN group, when compared with AIDP and HC groups (Fig. 1c–e). The ratio of (Tfh2+Tfh17)/Tfh1 was used to display skewed trend of the distribution of the three subsets. As expected, the ratio of (Tfh2+Tfh17)/Tfh1 was found raised in AMAN, but not in AIDP group (Fig. 1f). Collectively, these observations indicate a central role of Tfh2 and Tfh17 subsets in the pathogenesis of AMAN.

### Higher (Tfh2+Tfh17)/Tfh1 ratio in severe disease

**Figure 2:**
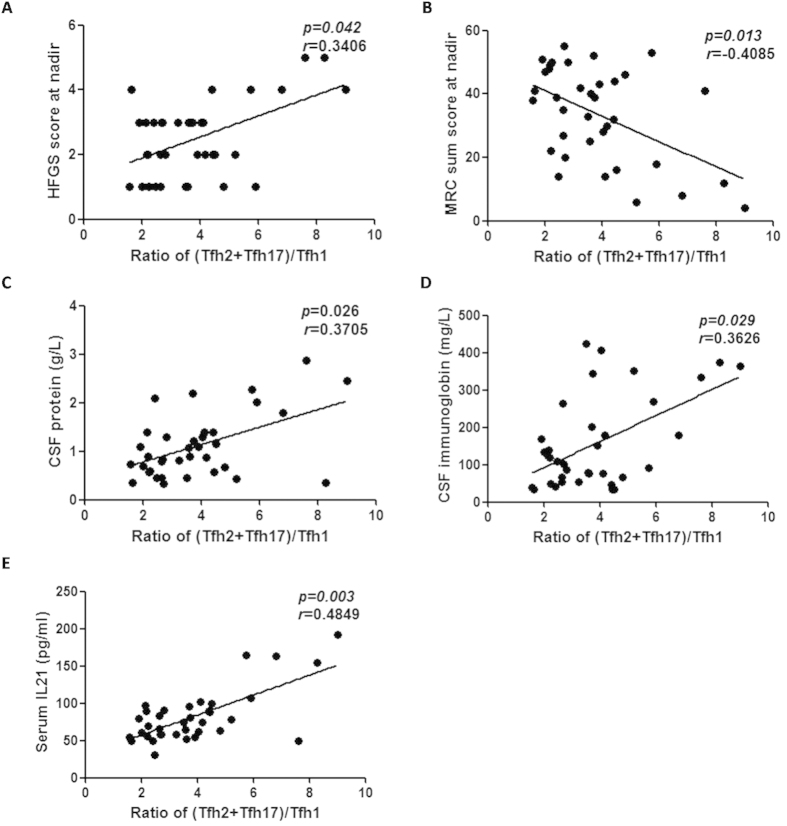
Correlation of clinicopathological features of GBS with the (Tfh2+Tfh17)/ Tfh1 ratio. (**A**) Positive correlation of Hughes Functional Grading Scale score (HFGSs) at nadir with (Tfh2+Tfh17)/Tfh1 ratio. (**B**) Negative correlation of the Medical Research Council sum score (MRC sum score) at nadir with the (Tfh2+Tfh17)/Tfh1 ratio. (**C**,**D**) Positive correlation of CSF protein and immunoglobin levels with (Tfh2+Tfh17)/Tfh1 ratio. (**E**) Positive correlation of serum IL-21 concentration with (Tfh2+Tfh17)/Tfh1 ratio.

Previous studies have demonstrated that the two Tfh subsets, Tfh2 and Tfh17 secrete IL-21^16^, which is known to play a crucial role in the expansion and plasma cell differentiation of co-cultured B cells^24^. As is already known, the abnormal distribution of the circulating memory Tfh cell subsets had a robust association with the pathogenesis of several autoimmune diseases that manifested abnormal humoral immune system^16^,^18^,^25^. However, there is paucity of similar evidence in the case of GBS. In our study, a positive correlation was observed between the levels of Tfh2 and Tfh17 subsets, and the severity of clinicopathological features of GBS. The ratio of (Tfh2+Tfh17)/Tfh1 showed a positive correlation with the HFGS score, and a negative correlation with the MRC sum score (Fig. 2a,b). There was also a positive correlation between the ratio of (Tfh2+Tfh17)/Tfh1 and the concentration of CSF total protein and CSF immunoglobin levels (Fig. 2c,d); Consistent with earlier studies^16^, the higher counts of Tfh2 and Tfh17 subsets showed a positive correlation with the serum IL-21 levels (Fig. 2e).

### Correlation of (Tfh2+Tfh17)/Tfh1 ratio with absolute plasmablasts count

**Figure 3:**
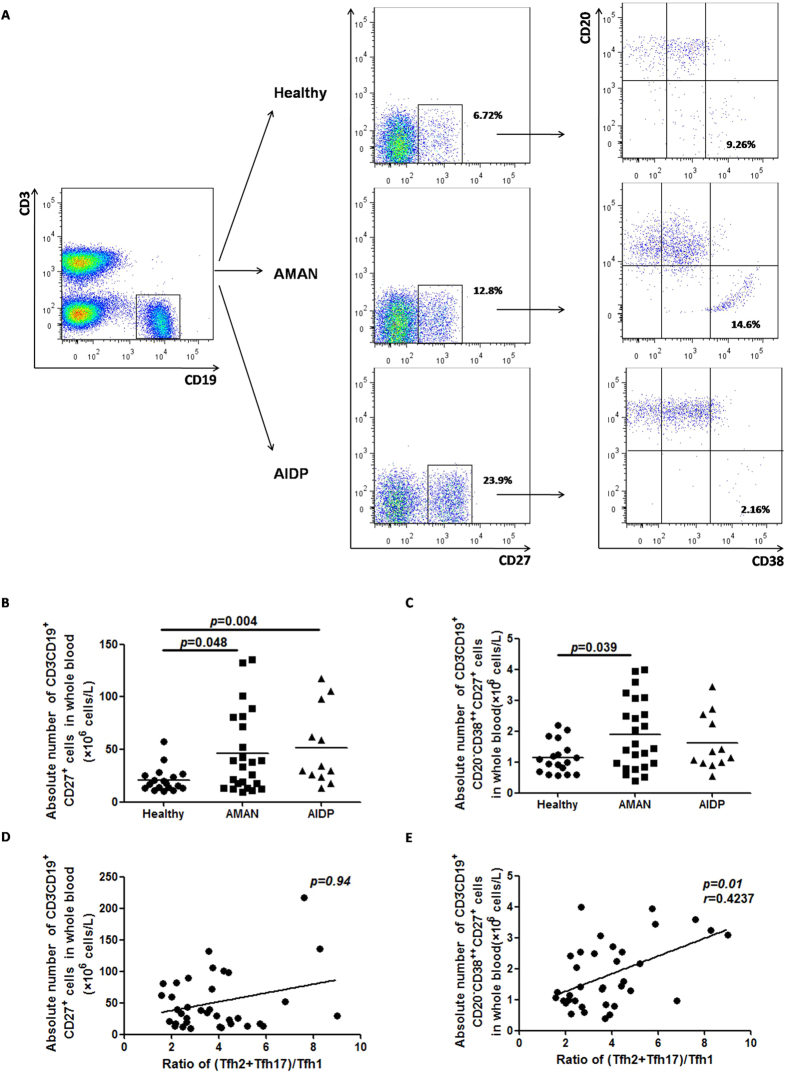
Correlation of skewed distribution of circulating memory Tfh subsets with plasmablast alteration. (**A**) PBMCs from GBS patients as well as HC were stained with anti-CD3, anti-CD19, anti-CD27, anti-CD20, and anti-38. The cells were gated initially on living lymphocytes followed by on CD3−CD19+ B cells (**A**, top left). The frequency of CD27+ memory B cells and CD27+CD20–CD38 plasmablasts were analyzed by flow cytometry. At least 30,000 events were analyzed per sample. Data are representatives of different groups of samples from at least two independent experiments. (**B**,**C**) Quantification of total memory B cells and plasmablasts in whole blood are shown as absolute numbers and expressed as cells/L of individual samples from at least two separate experiments. Findings of Mann Whitney test. The horizontal lines represent the median values. (**D**,**E**) Frequency of plasmablasts, but not total memory B cells, positively correlated with (Tfh2+Tfh17)/Tfh1 ratio.

Considering the high proportion of Tfh2 and Tfh17 subsets in AMAN, we predicted a role of aberrant humoral immunity in the pathogenesis of AMAN. We determined the absolute peripheral blood count of total memory B cells (CD3^−^CD19^+^CD27^+^) and plasmablasts (CD3^−^CD19^+^CD27^+^CD38^high^CD20^−^) in AMAN, AIDP and HC (Fig. 3a), and found that the absolute count of total memory B cells in patients with AMAN and AIDP were higher than that in the HC (Fig. 3b); while the absolute count of plasmablasts was raised only in the AMAN group (Fig. 3c). On correlation analysis, the ratio of (Tfh2+Tfh17)/Tfh1 did not significantly correlate with the absolute count of total memory B cells (Fig. 3d), but there was a positive statistical correlation with the absolute count of plasmablasts (Fig. 3e). These results indicate that Tfh2 and Tfh17 subsets may have a role in the pathogenesis of AMAN by assisting in terminal differentiation of B cells and yielding more antibody-producing cells.

### Elevated ICOS^+^ cells in Tfh2 and Tfh17 cells, and the inverse in PD-1^+^ cells

**Figure 4:**
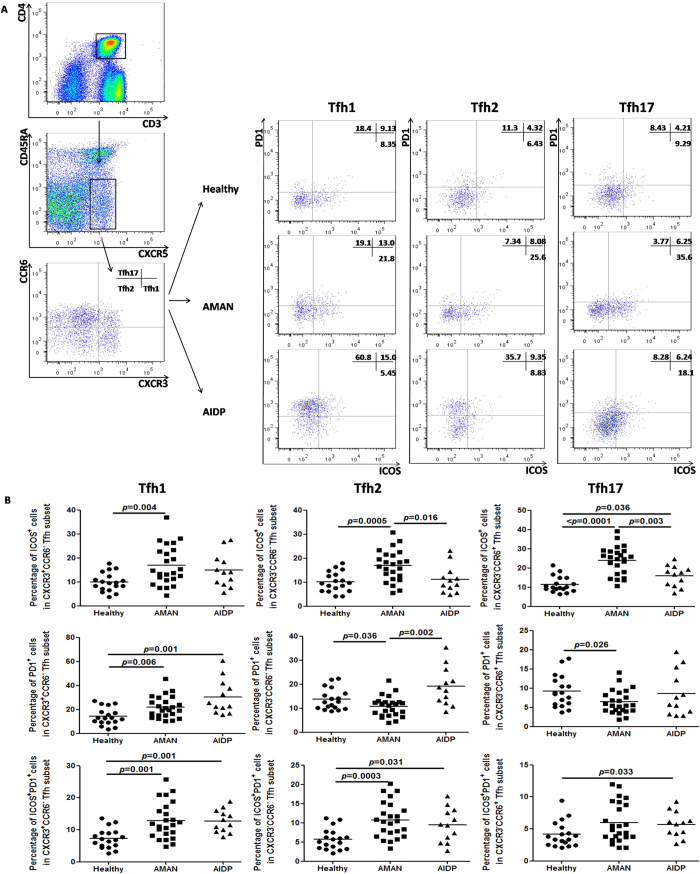
Differential distribution of ICOS+, PD-1+ and ICOS+PD-1+ cells in Tfh cell subsets (Tfh1, Tfh2, and Tfh17). (**A**) Tfh cell subsets from GBS patients and HC were stained with anti-CD278 (ICOS), anti-CD279 (PD-1). The percentages of ICOS^+^ cells, PD-1^+^ cells and ICOS^+^ PD-1^+^cells were assessed by flow cytometry. At least 30,000 events were analyzed for each sample. Data are representatives of different groups of samples from at least two independent experiments. (**B**) The percentages of ICOS^+^ cells, PD-1^+^ cells and ICOS^+^PD-1^+^ cells among Tfh1, Tfh2, and Tfh17 cells were respectively analyzed by Mann Whitney test. The horizontal lines show the median values.

For identifying the differential expression of general surface markers on the circulating memory Tfh cell subsets, and for exploring whether these changes affect the cellular function, we screened the expression of ICOS and PD-1 on circulating memory Tfh cell subsets in AMAN, AIDP and HC patients. ICOS is a well-known molecule that contributes in inducing the production of IL-21 by Tfh cells, and also significantly supports the synthesis of immunoglobulins^15^,^26^. Conversely, the main role of PD-1 in normal human physiology is to suppress autoimmunity by acting as a co-inhibitory immune checkpoint^27^. Thus, we postulate a role of the percentages of ICOS^+^ cells, PD-1^+^ cells and ICOS^+^PD-1^+^ cells in deciding the expression of circulating memory Tfh cell subsets in patients. As shown in Fig. 4b, the percentage of ICOS^+^ cells was raised in all the three Tfh subsets in the AMAN group. In particular, the percentage of ICOS^+^ cells in Tfh2 and Tfh17 subsets was significantly higher than that in the AIDP group. However, only the percentage of PD-1^+^ cells in Tfh2 and Tfh17 cells in the AMAN group was found to be statistically lower when compared with that in the HC group. Moreover, there was statistically significant difference between the percentages of PD-1^+^cells and ICOS^+^PD-1^+^ cells in Tfh1 and the percentages of ICOS^+^PD-1^+^ cells in Tfh2 among AMAN and HC groups. These findings indicate that Tfh2 and Tfh17 subsets are strongly activated in AMAN patients.

### Correlation of Tfh2 and Tfh17 subset activation with autoantibody-related humoral immune response in AMAN

**Figure 5:**
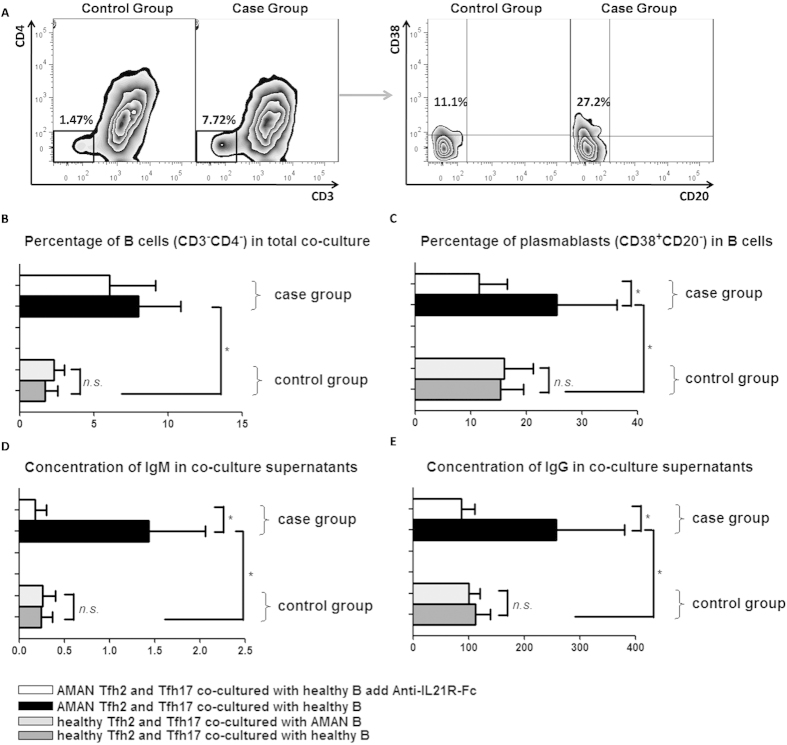
Circulating memory Tfh2 and Tfh17 subsets from AMAN patients induce allogenic normal B cells to differentiate into more Ig-producing plasmablasts. (**A**) PBMCs from severe AMAN patients and HC were stained with anti-CD3, anti-CD4, anti-CXCR5, anti-CD45RA, anti-CXCR3 anti-CCR6 and anti-CD19. Sorted circulating memory Tfh subsets (Tfh2 and Tfh17) and B cells, and then co-cultured them for 6 days. Circulating memory Tfh2 and Tfh17 subsets from severe AMAN patients (case group) or healthy donor (control group) were cultured with allogenic B cells, collected total mixed cells at day 6 and analyzed by FACS. CD3-CD4- survival B cells and CD38+CD20- plasmablast populations are representative of different groups of samples from at least two independent experiments. (**B**,**C**) Comparison of the percentages of survival B cells in the cultures and the percentage of plasmablasts within B cells, at day 6 between case and control groups. (**D**,**E**) Comparison IgM and IgG concentrations in the cultures at day 6 between case and control groups. Data analysis by Mann Whitney test and expressed as Median ± SD. Statistical significance was indicated by *P* < 0.05.

To explore the effect of Tfh2 and Tfh17 subsets on B cells terminal differentiation and isotype switching, we designed an *in vitro* co-culture system. Representative flow cytometry dot plots of the results obtained are shown in Fig. 5a. In order to exclude the effect of over active B cells on humoral immunity, two control groups were set up, which included HC derived Tfh2 and Tfh17 cells co-cultured with HC derived B cells, and HC derived Tfh2 and Tfh17 cells co-cultured with AMAN derived B cells. As expected, no statistical differences was observed between the two control groups (Fig. 5b–e). The case group included AMAN derived Tfh2 and Tfh17 cells co-cultured with HC derived B cells, and AMAN patient derived Tfh2 and Tfh17 co-cultured with HC derived B cells, with added 1μg/mL soluble rabbit antihuman-IL-21R-Fc (Peprotech, Rocky Hill, NJ). On comparison of the two case groups, we found that the blockade of IL-21 with soluble rabbit antihuman-IL-21R-Fc resulted in significantly suppressed co-cultured B cell differentiation and immunoglobin IgG and IgM production (Fig. 5c–e). Moreover, compared with HC, the percentages of viable B cells (CD3–CD4-) in the culture, and the percentage of plasmablasts (CD38+CD20-) within B cells at day 6, were all raised to a higher level in the case group (Fig. 5b,c), with a concomitant increase in concentration of IgM and IgG in co-culture supernatants (Fig. 5d,e). Collectively, these observations indicate that Tfh2 and Tfh17 subsets from AMAN patients displayed a higher efficiency to maintain B cell survival and promote their differentiation into antibody-secreting cells, and that the inducing effect was partly inhibited by blocking of IL21.

### Decreased circulating memory Tfh cells, but not plasmablasts after IVIg treatment

**Figure 6:**
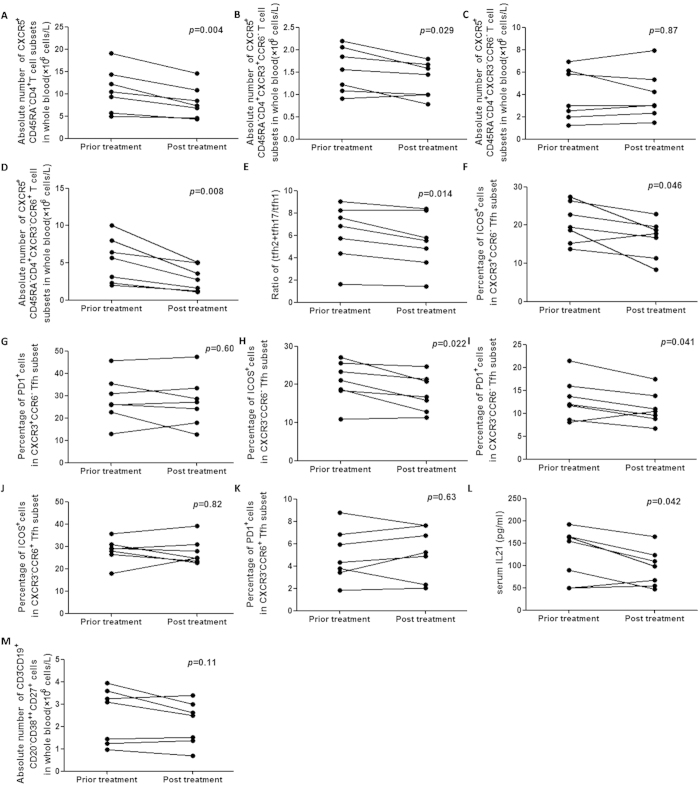
Treatment with high-dose IVIg modulates the frequency of total Tfh cells in severe AMAN patients, followed by the alteration of serum IL-21 and (Tfh2+Tfh17)/Tfh1 ratio. A total of 7 severe AMAN patients were treated with high-dose IVIg for 1 week. (**A–D**) comparisons of the frequency of total Tfh cells and their three subsets (Tfh1, Tfh2 and Tfh17); (**E**) (Tfh2+Tfh17)/Tfh1 ratio; (**F**–**K**) the percentages of ICOS^+^ cells and PD-1^+^ cells in Tfh subsets; (**L**) serum IL-21 concentration and (**M**) the frequency of plasmablasts between IVIg pre- and post treatment. Data analyzed by Paired *t* test.

To understand the effect of IVIg treatment on modulation of the autoimmune state in severe AMAN, we determined the counts of the total circulating memory Tfh cells and their three subsets (Tfh1, Tfh2 and Tfh17), the ratio of (Tfh2+Tfh17)/Tfh1, the level of serum IL-21 in the seven patients who had severe disease (HFGSs ≥4). Furthermore, we also determined the absolute number of plasmablasts and the percentages of ICOS^+^ and PD-1^+^ cells in Tfh1, Tfh2 and Tfh17 subsets. We noted that after IVIg treatment, the frequency of total Tfh cells dropped (Fig. 6a), with a concomitant decline in the ratio of (Tfh2+Tfh17)/Tfh1 (Fig. 6e) and serum IL-21 levels (Fig. 6l). Among them Tfh17 decreased the most obviously, followed by Tfh1, while Tfh2 did not significantly decrease (Fig. 6b–d). However, as to the percentages of ICOS^+^ and PD-1^+^ cells in Tfh subsets after treatment, significantly decreased indicators only include ICOS^+^ cells in Tfh1 and ICOS^+^ and PD-1^+^ cells in Tfh2 (Fig. 6f,h,i). Moreover, the number of plasmablasts were similar in the same patient, before and after IVIg treatment (Fig. 6m). These results indicate the efficacy of IVIg treatment in effecting a reduction in the number of Tfh cells, but that the treatment was not capable of deactivating these cell subsets.

## Discussion

In the present study, phenotypic and functional characterization of circulating memory Tfh cells in 36 GBS patients (including AMAN (N = 24) and AIDP (N = 12) patients was performed by flow cytometry, cell-sorting and co-culture techniques. We found that almost all of the patients with severe HFGS score (≥4) belonged to the AMAN group. Similar to our results, Zhang, *et al.* also reported more severe clinical symptoms and poorer short-term prognosis in the antiganglioside antibody-associated GBS, which mainly presents with acute motor axonal injury^14^. Immunopathogenesis of GBS is thought to involve: (i) CD4^+^Th-cell-mediated response against myelin antigens, and, (ii) anti-ganglioside antibody and activated complement components induced axon damage^1^. It is speculated that T-B cell interactions which are known to induce excessive humoral immune response may be the fundamental aspect of immunopathogenesis in the severe cases.

Some studies reported that circulating Tfh cells shared phenotypic and functional properties with their GC counterparts^16^, and that they are probably derived from GC-Tfh cells^28^. The rapid increase in their memory subsets in peripheral circulation under auto-antigenic stimulation can potentially serve as biomarkers for monitoring active GC response in lesions^29^.

Although the exact molecular mechanism of circulating memory Tfh-B cell axis remains to be elucidated, various studies have indicated a pivotal role of Tfh cells in amplifying autoreactive B cells and promoting production of autoantibodies, which eventually leads to a cascade of pathological changes in certain autoimmune diseases^16^,^19^,^20^,^29^,^30^,^31^. Our findings indicate that the elevated counts of Tfh2 and Tfh17 cells were particularly related to more severe clinical symptoms, and that it showed a positive correlation with the levels of plasmablasts in GBS patients. In other words, Tfh2 and Tfh17 subsets promote the skewed expression of B cells towards antibody-producing cells, which leads to increased antibody-mediated humoral immune response.

To explore the potential relationship between the phenotypic characteristics and the functional properties of Tfh2 and Tfh17 subsets in AMAN patients, we detected the expression of general surface markers, such as ICOS and PD1. ICOS is known to facilitate the production of IL-21 by Tfh cells, and the synthesis of immunoglobulins^15^,^26^. Recent studies have documented higher expression of ICOS and ICOS-L mRNA in sural nerve biopsy specimens of GBS patients^32^. Conversely, in normal human physiology, the main role of PD1 is to suppress autoimmunity by acting as a co-inhibitory immune checkpoint expressed on the surface of various immunological cells^27^. Further, the PD1-PDL pathway is known to thwart self-reactive T cells and protect against autoimmune pathology within the CNS^33^,^34^. Others have suggested that T cells with deficient PD-1 signaling may be preferentially polarized towards effector T-cell differentiation^35^. Therefore, we hypothesize that the expression of ICOS and PD-1 may determine the immunological status (whether suppressed or activated) of circulating memory Tfh cells in GBS patients.

We also observed an increased percentage of ICOS^+^ cells and decreased percentage of PD-1^+^ cells in Tfh2 and Tfh17 subsets in AMAN patients, which indicates that patients with AMAN not only have increased numbers of circulating memory Tfh2 and Tfh17 subsets, but are also over-activated, which is consistent with the abnormal humoral immune response. Subsequently, the functional *in vitro* experiments in our study verified that Tfh2 and Tfh17 subsets in AMAN patients possessed stronger activity with regards to maintaining B cell survival and promoting B cells differentiation into antibody-secreting cells. This effect was found to be partly inhibited by blockage of IL21.

Undoubtedly, as a traditional first line therapy, high-dose IVIg treatment is effective in a number of auto-immune disorders by down-regulating autoreactive T/B cell function^36^. So far, the effect of IVIg treatment on Tfh cell differentiation and function has not been explored. In the present study, our data, perhaps for the first time, reveals that high-dose IVIg treatment down-regulates the absolute counts of circulating memory Tfh cells in serious GBS (HFGSs ≧4) patients, which was associated with the decline in the (Tfh2+Tfh17)/Tfh1 ratio along with a decline in serum IL-21 levels. However, the precise mechanism of action of the same is not completely understood. The mechanism of action of IVIG in GBS has earlier been shown to include a direct competition with autoantibodies, by neutralization of autoantibodies by anti-idiotypic antibodies^37^, and by inhibition of complement deposition^38^. A recent study reported downregulation of circulating memory CD4^+^T cells and CD19^+^ B cells in AIDP, but not in AMAN, among GBS patients treated with IVIg^39^. However, the downregulation of circulating CD4^+^T cell subsets, Th1, Th17 and Th22, was not related with GBS subtypes^40^. Furthermore, we found no significant difference in the percentage of ICOS^+^ and PD-1^+^ cells among Tfh17 and the number of plasmablasts between pretreatment and posttreatment in patients with severe AMAN, which indicates a less than satisfactory efficacy of traditional IVIg in affording symptom-relief in patients with severe AMAN, probably due to the poor modulation of Tfh-B cell interactions.

## Conclusion

To the best of our knowledge, this is the first study to demonstrate the association of circulating memory Tfh cells, especially the Tfh2 and Tfh17 subsets, with the development of AMAN. Not only were the absolute counts of circulating memory Tfh2 and Tfh17 subsets elevated in peripheral blood of AMAN patients, but these cells were also in an overactive state, which in turn stimulated the antibody-mediated humoral immune response, leading to more severe disease manifestations in AMAN patients.

Our study indicates that peripheral Tfh2 and Tfh17 cell counts may serve as useful bio-markers for evaluating the disease progression in AMAN patients. Additionally, these cells may have future application as therapeutic targets for the treatment of AMAN patients.

## Additional Information

**How to cite this article**: Che, Y. *et al.* Circulating memory T follicular helper subsets, Tfh2 and Tfh17, participate in the pathogenesis of Guillain-Barré syndrome. *Sci. Rep.* **6**, 20963; doi: 10.1038/srep20963 (2016).

## Figures and Tables

**Table 1:** Baseline characteristics of patients with Guillain-Barré syndrome (GBS) and Healthy controls (HC).

Parameters	GBS	HC	*P*-value
Number	36	18	NS
Age, years (range)	46.03 (19–78)	47.08 (27–59)	NS
Gender (F/M)	12/24	7/11	NS
WBC (×10^9^/L)	7.6 (4.3–12.8)	6.5 (4.1–8.9)	NS
Lymphocytes (×10^9^/L)	1.94 (1.41–2.74)	1.67 (1.18–2.12)	NS
WBC in CSF (×10^6^/L)	7 (2–11)	5 (0–8)	NS
CSF Immunoglobins (mg/L)	157.5 (35.4–425)	22 (0–34)	<0.01
CSF protein level (g/L)	1.1 (2.88–0.35)	0.35 (0–0.46)	<0.01

WBC,White blood cells; CSF, cerebrospinal fluid.Reference values: WBC (3.5–9.5) × 10^9^/L; Lymphocytes (1.1–3.2) × 10^9^/L; WBC in CSF: (0–8)  × 10^6^/L; Immunoglobin in CSF (0–34) mg/L; Protein in CSF (0.15–0.45) g/L.

**Table 2:** Clinical characteristics of GBS patients.

No.	Sex	Age (years)	Previous infections	Electrophysiological findings	HFGSs at nadir	MRC sum score at nadir
1	Male	42	GI	AMAN	1	27
2	Female	50	GI	AMAN	3	40
3	Male	57	None	AIDP	3	48
4	Male	27	GI	AMAN	2	22
5	Female	26	URTI	AMAN	3	39
6	Male	63	URTI	AMAN	4	53
7	Male	19	GI	AMAN	5	12
8	Male	74	None	AMAN	4	8
9	Male	23	None	AIDP	3	28
10	Male	78	URTI	AIDP	1	50
11	Male	48	None	AMAN	2	16
12	Male	28	URTI	AMAN	2	50
13	Female	29	None	AMAN	4	32
14	Male	46	GI	AMAN	3	14
15	Male	52	URTI/GI	AMAN	2	6
16	Male	41	GI	AIDP	1	25
17	Male	42	URTI	AMAN	3	42
18	Male	60	None	AMAN	3	52
19	Female	29	URTI	AIDP	2	44
20	Female	53	GI	AMAN	1	33
21	Male	34	URTI	AIDP	2	35
22	Female	49	URTI/GI	AMAN	3	20
23	Female	28	None	AIDP	2	49
24	Male	24	URTI	AMAN	3	39
25	Female	41	URTI/GI	AMAN	3	55
26	Female	32	GI	AMAN	2	43
27	Female	46	URTI	AIDP	2	30
28	Female	60	None	AIDP	1	47
29	Male	54	None	AIDP	1	38
30	Male	36	None	AMAN	5	41
31	Male	39	None	AIDP	3	51
32	Male	61	None	AMAN	4	4
33	Male	61	URTI/GI	AMAN	1	14
34	Female	65	None	AMAN	1	46
35	Female	64	URTI	AMAN	4	41
36	Female	76	URTI	AIDP	1	18

HFGSs, Hughes Functional Grading Scale score; MRC sum score, Medical Research Council sum score; GI, gastrointestinal infections; URTI, upper respiratory tract infections; AIDP, acute inflammatory demyelinating polyradiculoneuropathy; AMAN, acute motor axonal neuropathy.

**Table 3:** Laboratory data of GBS patients.

No.	HFGSs at nadir	MRC sum score at nadir	Memory B (×10^6^ cells/L)	Plasmablast (×10^6^ cells/L)	Tfh1 (×10^6^ cells/L)	Tfh2 (×10^6^ cells/L)	Tfh17 (×10^6^ cells/L)	Ratio of (Tfh2+Tfh17/Tfh1)	Serum IL-21 (pg/mL)	CSF Ig (mg/L)	CSF protein (g/L)
1	1	27	25.74	2.53	2.36	2.18	4.05	2.6398305	83.44	54	0.8
2	3	40	39.41	1.4	2.65	4.72	4.84	3.6075472	52.52	76	0.9
3	3	48	13.02	1	2.32	1.88	3.07	2.1336207	97.21	123	1.39
4	2	22	81.82	2.41	2.66	2.57	3.27	2.1954887	56.02	118	0.57
5	3	39	33.54	0.96	2.25	2.39	3.04	2.4133333	49.85	41.2	2.1
6	4	53	17.47	3.95	1.56	5.81	3.15	5.7435897	164.33	92	2.28
7	5	12	135.82	3.23	2.06	6.95	10.05	8.2524272	154.60	373	0.35
8	4	8	52	0.97	1.84	6.13	6.4	6.8097826	162.76	179	1.79
9	3	28	12.61	2.72	1.57	3.61	2.71	4.0254777	62.10	406	1.3
10	1	50	39.2	0.54	3.25	3.01	4.3	2.2492308	70.09	50	0.6
11	2	16	17.5	1.6	2.41	1	9.84	4.4979253	100.07	34.9	1.16
12	2	50	9.75	0.58	2.33	5.55	1	2.8111588	90.74	87	1.3
13	4	32	98	1.43	0.91	2	2	4.3956044	89.11	47	1.4
14	3	14	11	0.8	0.89	1.65	2	4.1011236	101.55	76.8	1.4
15	2	6	13	2.17	1.79	7	2.29	5.1899441	78.03	352	0.43
16	1	25	132.45	1.34	0.75	1.97	0.7	3.56	65.02	78	1.07
17	3	42	38	2.5	3.03	3.99	5.79	3.2277228	58.95	55	0.81
18	3	52	72	0.4	1.92	3.07	4.01	3.6875	96.30	201	2.2
19	2	44	23.45	2.54	1.82	5.26	2.83	4.4450549	88.11	35.4	0.58
20	1	33	34.11	3.07	2.2	2.51	5.22	3.5136364	74.34	425	0.46
21	2	35	19.58	1.41	1.51	3.1	0.89	2.6423841	66.01	66	0.46
22	3	20	89	0.77	4.94	4.17	9.17	2.7004049	58.10	101	0.34
23	2	49	17.61	1.15	2.12	2.26	2.35	2.1745283	89.03	140	0.9
24	3	39	105.57	0.84	4.56	5	12	3.7280702	80.75	344	1.21
25	3	55	42.8	4	3.82	3.8	6.35	2.6570681	58.21	263	0.84
26	2	43	30	0.52	2.59	5.21	4.9	3.9034749	54.60	152	1.1
27	2	30	101	2.24	1.61	0.74	6	4.1863354	75.04	178	0.88
28	1	47	59	0.89	1.81	1.61	2	1.9944751	61.11	133	0.7
29	1	38	62.07	1.07	2.63	0.85	3.3	1.5779468	55.07	40.4	0.74
30	5	41	217.18	3.59	1.08	2.53	5.67	7.5925926	49.85	335	2.88
31	3	51	21.4	0.97	3.01	1.93	3.75	1.8870432	80.02	169	1.1
32	4	4	29.4	3.1	1.22	3	8	9.0163934	192.22	363	2.46
33	1	14	12.47	2.05	1.98	1.91	2.98	2.469697	31.11	109	0.46
34	1	46	25.8	1.3	2.1	2.46	7.6	4.7904762	63.54	67	0.68
35	4	41	80.9	1.25	2.2	1.25	2.32	1.6227273	50.01	35.4	0.35
36	1	18	13	3.44	0.49	1.32	1.56	5.877551	107.07	269	2.01

**Table 4:** Comparison of clinical features between AMAN and AIDP patients.

Variables	AMAN	AIDP	*P*-value
HFGS score at nadir	2.8 (1–5)	1.8 (1–3)	0.018
HFGS score at discharge	2.5 (0–4)	1.3 (1–2)	0.013
MRC sum score at nadir	31.2 (4–55)	38.6 (18–51)	0.28
MRC sum score at discharge	34.7 (4–55)	46.5 (20–53)	0.039
Ventilation ratio	(5/24) 20.8%	(1/12) 8.3%	0.34
Electrophysiological findings	Axonal damage	Demyelination	NS
Protein level in CSF (g/L)	1.18 (0.35–2.88)	0.98 (0.46–2.01)	0.71
Immunoglobulin in CSF (mg/L)	165.9 (35.4–425)	140.7 (35.4–406)	0.78

HFGS, Hughes Functional Grading Scale; MRC sum, Medical Research Council.
